# Methods to test the interactive effects of drought and plant invasion on ecosystem structure and function using complementary common garden and field experiments

**DOI:** 10.1002/ece3.2729

**Published:** 2017-02-05

**Authors:** Christina Alba, Julienne E. NeSmith, Catherine Fahey, Christine Angelini, Stephen Luke Flory

**Affiliations:** ^1^ Agronomy DepartmentUniversity of FloridaGainesvilleFLUSA; ^2^ School of Natural Resources and EnvironmentUniversity of FloridaGainesvilleFLUSA; ^3^ Department of Environmental Engineering SciencesEngineering School for Sustainable Infrastructure and EnvironmentUniversity of FloridaGainesvilleFLUSA

**Keywords:** biological invasions, climate change, environmental gradient, rainout shelter, soil moisture

## Abstract

Abiotic global change drivers affect ecosystem structure and function, but how they interact with biotic factors such as invasive plants is understudied. Such interactions may be additive, synergistic, or offsetting, and difficult to predict. We present methods to test the individual and interactive effects of drought and plant invasion on native ecosystems. We coupled a factorial common garden experiment containing resident communities exposed to drought (imposed with rainout shelters) and invasion with a field experiment where the invader was removed from sites spanning a natural soil moisture gradient. We detail treatments and their effects on abiotic conditions, including soil moisture, light, temperature, and humidity, which shape community and ecosystem responses. Ambient precipitation during the garden experiment exceeded historic norms despite severe drought in prior years. Soil moisture was 48% lower in drought than ambient plots, but the invader largely offset drought effects. Additionally, temperature and light were lower and humidity higher in invaded plots. Field sites spanned up to a 10‐fold range in soil moisture and up to a 2.5‐fold range in light availability. Invaded and resident vegetation did not differentially mediate soil moisture, unlike in the garden experiment. Herbicide effectively removed invaded and resident vegetation, with removal having site‐specific effects on soil moisture and light availability. However, light was generally higher in invader‐removal than control plots, whereas resident removal had less effect on light, similar to the garden experiment. Invasion mitigated a constellation of abiotic conditions associated with drought stress in the garden experiment. In the field, where other factors co‐varied, these patterns did not emerge. Still, neither experiment suggested that drought and invasion will have synergistic negative effects on ecosystems, although invasion can limit light availability. Coupling factorial garden experiments with field experiments across environmental gradients will be effective for predicting how multiple stressors interact in natural systems.

## 1 Introduction

Climate change and biological invasions are global change drivers that threaten ecosystem structure and function (Mack & Simberloff, 2000; Root, Price, Hall, & Schneider, 2003; Thuiller, Lavorel, Araújo, Sykes, & Prentice, 2005; Vitousek, D'Antonio, Loope, Rejmanek, & Westbrooks, 1997). Their interactive effects can be additive, synergistic, or offsetting, and are difficult to predict (Atkinson & Urwin, 2012; Bradley, Oppenheimer, & Wilcove, 2009; Côté, Darling, & Brown, 2016; Crain, Kroeker, & Halpern, 2008; Reich et al., 2014). Changes in precipitation, temperature, and CO_2_ can influence plant physiological processes, including those of non‐native invaders (Dukes & Mooney, 1999; Weltzin, Belote, & Sanders, 2003), and will likely scale up to mediate plant species distributions on the landscape (Allen & Breshears, 1998; Root et al., 2003; Walther, Beißner, & Burga, 2005). Likewise, as invaders spread, so does their potential for interactions with resident species, which themselves may shift ranges under novel conditions (Allen & Breshears, 1998; Walther et al., 2005; Weltzin, Belote, et al., 2003). However, field studies that have experimentally manipulated resident–invader interactions under potential climate change conditions are rare (English, Weltzin, Fravolini, Thomas, & Williams, 2005; Holzapfel & Vinebrooke, 2005).

Soil moisture is a major factor influencing plant growth and ecosystem functions (Hoover, Knapp, & Smith, 2014; Knapp et al., 2008; Weltzin, Loik, et al., 2003). In natural systems, drought can cause large scale and persistent shifts in species distributions, for example through differential mortality of drought‐tolerant versus drought‐intolerant species (Allen & Breshears, 1998; Engelbrecht et al., 2007; Mueller et al., 2005). Thus, predicted increases in regional drought due to climate change have spurred research on ecosystem responses to low‐moisture stress (He & Dijkstra, 2014; Ledger, Brown, Edwards, Milner, & Woodward, 2013). A common field approach to evaluate drought effects on individuals, communities, and ecosystems (Cherwin & Knapp, 2012; De Dios Miranda, Padttla, Lázaro, & Pugnaire, 2009; Fay, Carlisle, Knapp, Blair, & Collins, 2000) is to use rainout shelters that reduce or alter the frequency of precipitation (Erbs, Manderscheid, & Weigel, 2012; Fay et al., 2000; Knapp et al., 2016; Yahdjian & Sala, 2002).

Less well studied is how invasive species mediate drought‐associated physiological stress in native communities and ecosystems. Invaders can dominate communities through competition for limiting resources such as water, nutrients, or light (Funk & Vitousek, 2007; Weiner & Vila, 2004), or through rapid responses to disturbance (Daehler, 2003; Davis, Grime, & Thompson, 2000). The effects of invasive species may be more pronounced under drought conditions that reduce resistance of resident communities to invasion (Diez et al., 2012). However, invasive species also may facilitate resident species via habitat modifications that create more benign environmental conditions (Rodriguez, 2006). Invaders could thus offset drought‐associated abiotic stress by, for example, increasing humidity or decreasing temperature within their dense canopy, thereby reducing evaporative soil moisture loss.

Simultaneously manipulating multiple global change drivers may be the most effective method to gauge their effects, but such experiments can be logistically difficult and may have reduced realism. Most studies exploring how drivers interact have manipulated abiotic factors, such as CO_2_ concentrations, precipitation, and temperature (Dieleman et al., 2012; Dukes et al., 2005; Erbs et al., 2012), limiting the inferences that can be drawn about ecosystem responses to biotic components of global change (Beier et al., 2012). Improving our understanding of how abiotic and biotic drivers together affect ecosystems is critical because such interactions are common and may not be additive, but could be synergistic or offsetting (Atkinson & Urwin, 2012; Côté et al., 2016; Crain et al., 2008; Darling & Côté, 2008). For example, drought stress can increase or decrease resistance to insect and pathogen attack (Jactel et al., 2012), resulting in disparate outcomes that cannot be predicted by evaluating responses to stressors in isolation. Currently, only a few studies to our knowledge have simultaneously measured the effects of drought, invasion and their interactions on abiotic factors, organisms, and ecosystem functions (Caldeira et al., 2015; English et al., 2005; Huxman et al., 2004).

To assess the individual and interactive effects of drought and invasion, we paired a factorial common garden experiment composed of planted resident communities exposed to drought and invasion with an invasion removal experiment at field sites spanning a natural soil moisture gradient. Here, we detail how to implement this dual approach, which provides a comprehensive and mechanistic test of drought and invasion effects while broadening inference to the landscape scale (Kayler et al., 2015) and report on how experimental drought and invasion treatments affect the physical environment. The experiments were conducted in north‐central Florida, United States (US), with native pine tree species, a diversity of native and naturalized resident understory herbaceous plant species, and a non‐native invasive grass (cogongrass, *Imperata cylindrica*). This study system has broad conservation value because forests are particularly susceptible to heat and drought stress, with stand‐scale die‐offs occurring worldwide, including in the Southeast US (Allen et al., 2009). Whether pine forest ecosystems are more susceptible to the effects of plant invasions when they are under drought stress is unknown.

## 2 Materials and Methods

### 2.1 Study system

Pine forests of the Southeast US harbor very diverse plant communities but have been reduced from ~37 million to 1.5 million hectares since the arrival of European settlers (Frost, 1993). Both drought (Wang, Fu, Kumar, & Li, 2010) and invasions (Simberloff, Schmitz, & Brown, 1997; van Kleunen, Dawson, Essl, & Pergl, 2015) are intensifying in the region. Changes in soil water dynamics may represent a novel stressor to Southeast US plant communities. Specifically, fewer light to medium rainfall events in dry years have led to more severe summer drought in the region (Wang et al., 2010). Separately, invasion by cogongrass, a rhizomatous C4 grass introduced to the Southeast US from Asia in the early 1900s, also threatens pine forests. Cogongrass invades habitats ranging from intact forest understories to managed pastures and pine plantations (Dozier, Gaffney, Mcdonald, Johnson, & Shilling, 1998; King & Grace, 2000) and grows over a range of light, soil nutrient, and soil moisture conditions (Bryson, Krutz, Ervin, Reddy, & Byrd, 2010; Holzmueller & Jose, 2011). It covers more than 100,000 ha across the Southeast US (Estrada & Flory, 2015), where it often forms expansive monocultures that can drastically reduce light availability and native plant diversity in longleaf pine forests (Brewer, 2008). If cogongrass is better adapted to or can more efficiently alter trait expression in response to drought than resident plants, it may interact synergistically with drought stress to suppress pine trees and associated herbaceous species, potentially altering ecosystem functions. Alternatively, if cogongrass mitigates low soil moisture, it could buffer some species against drought stress.

### 2.2 Factorial common garden experiment

We conducted a factorial experiment to examine the effects of drought and invasion on physical environmental factors, pine performance, herbaceous understory and arthropod community structure, and ecosystem function. We note that this experiment *simulates* drought, a complex climatological phenomenon, by reducing precipitation via rainout shelters (hereafter, “drought”). We further note that while precipitation extremes (including unusually wet periods that punctuate drought) are also of interest (Knapp et al., 2016), we focused on drought to maximize our power to uncover the hypothesized drought and invasion interactions. Although the experiments are ongoing as of 2016, we use past tense language for clarity. Additionally, because the ecological outcomes are still emerging, our purpose here is to report on the methodology and the multi‐faceted physical responses we observed to the experimental treatments. We implemented 10 replicates of four treatments (4 m × 4 m plots, 40 total) in a randomized block design: ambient precipitation with resident understory species; drought with resident species; ambient precipitation with resident species plus the invader; and drought with resident species plus the invader. The experiment was located in an old field at the University of Florida Bivens Arm Research Site (BARS) in Gainesville, FL, US (29°37′42.4″N, 82°21′14.4″W; elevation 20 m a.s.l.), within the humid subtropical (Cfa) climate type of the Köppen Climate Classification system (Kottek, Grieser, Beck, Rudolf, & Rubel, 2006). Mean annual temperatures are between 13.9 and 27.2°C, and mean annual precipitation is 113 cm (Western Regional Climate Center, weather station ID 083316), with a dry season from October/November to April/May. Soils are primarily Portsmouth sandy loam (67% sand, 3% silt, and 30% clay) composed of Blichton sand (25%; 2%–5% slope) and Bivans sand (75%; 5%–8% slope; Natural Resources Conservation Service, Web Soil Survey).

To establish resident communities, we planted bareroot longleaf pine seedlings (*N* = 20 per plot, Florida Forest Service, Chiefland, FL, USA) and native herbaceous understory seedlings (12 native species × 3 individuals/species = 36 individuals per plot, The Natives Inc., Davenport, FL, USA) into the 40 field plots. Herbaceous seed was germinated in growth chambers and seedlings were transplanted after 8 weeks to containers (Steuwe & Sons, Inc., Tangent, OR, USA) where they grew for another 8 weeks in a greenhouse. Longleaf pine and herbaceous seedlings were transplanted into plots in May 2012 and watered until establishment. Seedlings that did not survive transplantation were replaced. Many other native and non‐native (but not invasive) species colonized the experiment from the seed bank and the surrounding area.

#### 2.2.1 Experimental treatments

**Figure 1:**
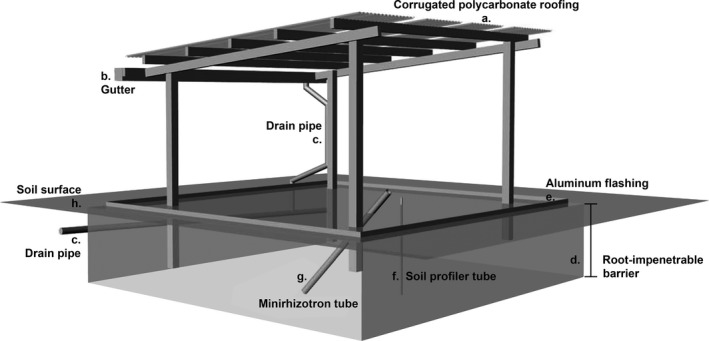
Schematic of the rainout shelter design for the common garden experiment

After resident plants established, we imposed drought starting in March 2013 and invasion in June 2013. To apply the drought treatment, we constructed rainout shelters that reduced precipitation by 89% (Figure 1). The shelters were constructed with pressure‐treated lumber and corrugated polycarbonate roof panels (89% light transmittance; TUFTEX PolyCarb; Figure 1a) or shade cloth (22% density; Greenhouse Megastore) as a control for rainout shelter light reduction. Precipitation runoff was collected by aluminum gutters (Amorfill Aluminum, Trenton, FL, USA; Figure 1b) and moved offsite via downspouts and drainage pipes (Figure 1c). Sub‐surface and surface water flow was diverted from drought plots by in‐ground and ground‐surface barriers. The in‐ground barriers were root‐impenetrable black plastic sheeting (Global Plastic Sheeting, Vista, CA, USA; Figure 1d) buried 1‐m deep around each plot. The plot perimeters were lined aboveground by aluminum flashing (Amerimax aluminum flashing; Figure 1e) buried to ~5 cm deep and extending ~10 cm above the soil surface. Each plot had separate tubes that accommodated a soil moisture profile probe (Figure 1f) and a minirhizotron (Figure 1g). In May 2013, we harvested cogongrass rhizomes from a single population at BARS and planted them in trays in the BARS greenhouse. After 3 weeks, ramets were separated and transplanted to plastic pots (5.1 × 5.1 × 7.6 cm). After three additional weeks, nine seedlings (~20 cm tall) were added to each invasion treatment plot on a 3 × 3 grid at 1‐m spacing. All seedlings survived transplantation.

#### 2.2.2 Data collection

We collected data on abiotic factors including volumetric water content (hereafter soil moisture), photosynthetically active radiation (hereafter light availability), humidity, and temperature. We measured soil moisture with two approaches: (1) bi‐monthly during the wet season and monthly during the dry season using a 12‐cm probe (*N* = 4 sub‐samples per plot, HydroSense II; Campbell Scientific) and periodically by inserting a PR2 profiler probe connected to an HH2 data logger into the profile tube in each plot (Dynamax, Houston, TX, USA) to simultaneously collect data at six depths (100, 200, 300, 400, 600, and 1000 mm). Light was measured monthly using an ACCUPAR LP‐80 linear ceptometer (Decagon Devices, Pullman, WA, USA). To quantify shelter effects on light availability, we measured light both outside and underneath shelters, above the plant canopy. To measure the effect of drought and invasion on light availability, we took measurements at 0.5 m (mid‐canopy) and ground level (below the canopy). Temperature and humidity were measured hourly at ground level at the center of each plot with Hygrochron™ ibutton sensors (model DS1923; Embedded Data Systems, LLC; Lawrenceburg, KY, USA) over 6 weeks during the wet and dry seasons. Shelter edge effects on temperature and humidity also were logged (see Appendix S1 for methods and results).

We measured (but do not report herein) the responses of pine trees, herbaceous plants, arthropods, and soil microbes to the drought and invasion treatments. Pine survival and performance (height and root collar diameter) were measured each February when seedlings were easy to locate and not subjected to stem‐swelling rains. Multiple years of data allowed us to track tree response to stress across ontogenetic stages that may exhibit different levels of susceptibility to drought and invasion, including the seedling, grass, and sapling tree life‐history stages. We quantified percent cover of the invader early and late in the growing season and aboveground biomass, tiller number, density, and height at peak biomass. We also evaluated resident understory (planted species and recruits) percent cover, species richness, diversity, and evenness, and aboveground biomass. Finally, we quantified the abundance and diversity of soil microbes, carbon and nitrogen cycling, canopy arthropod communities, and herbivory.

#### 2.2.3 Statistical analysis

We used a *t* test to evaluate effects of rainout and control shelters on light availability. To assess drought and invasion effects on soil moisture, light availability, temperature, and humidity, we used mixed model ANOVA. Separate models were used to evaluate treatment effects on light at 0.5 m and at ground level and on treatment effects on temperature and humidity during the wet and dry seasons. Fixed effects for all common garden models were drought, invasion, date, and their interactions. Block was treated as a random effect and plot as a repeated measure across dates (autoregressive covariance structure). Square‐root‐transformation improved normality and homogeneity of variance for soil moisture, light availability, and temperature during the wet season. Analysis of shelter edge effects on temperature and humidity is outlined in Appendix S1. All analyses were conducted in SAS v. 9.4 (SAS Institute, Cary, NC, USA).

### 2.3 Field experiment along natural soil moisture gradient

The field experiment was conducted at nine sites in north‐central Florida that varied in soil moisture due to their positioning in excessively well‐drained upland sand hill to poorly drained flatwood habitats (Appendix S2). All sites had established cogongrass invasions in stands of longleaf or slash pine. At each site, we established 4 m × 4 m plots, separated by at least 3 m, in invaded areas and in nearby control areas dominated by resident vegetation. Vegetation in invaded and control plots was left intact as a reference or removed (four treatments × three replicates, *N* = 12 plots per site, 108 total plots). Bareroot longleaf pine seedlings (*N* = 4 per plot) were planted in early 2015 and replaced within 8 weeks if they did not survive transplantation.

#### 2.3.1 Experimental treatments

Vegetation was initially removed from treated plots with a gas‐powered hedge trimmer (Andreas Stihl AG and Co. KG, Germany) and raking, and plant material was removed from plots. To maintain removal, we applied herbicide every 2–4 months when percent cover reached 10%–30%. We used broad‐spectrum glyphosate (Roundup Weathermax with active ingredient *N*‐(phosphonomethyl)glycine, Monsanto) at 6.165 kg active ingredient/ha with a CO_2_‐pressurized backpack sprayer calibrated to deliver 187 L/ha (20 g active ingredient/acre). Experimental pines were covered during herbicide applications.

#### 2.3.2 Data collection

To determine vegetation removal efficacy, we evaluated percent cover and biomass of native and invader vegetation across treatments. We also characterized soil moisture and light in all plots in September 2015 and June 2016. We collected data on pines, microbes, and nutrient cycling as in the common garden experiment. We characterized overstory pine stand characteristics associated with forest age structure, including tree canopy cover using a convex spherical densiometer (Model A; Forestry Suppliers, Inc., Jackson, MS, USA) and overstory tree DBH (diameter at breast height, 1.4 m above the ground) because these factors may co‐vary with soil moisture and influence vegetation characteristics and tree seedling responses to invasion along the gradient.

#### 2.3.3 Statistical analysis

To assess removal efficacy in terms of percent cover and biomass, and whether efficacy varied by site, we used two‐way ANOVA with site, treatment, and a site × treatment interaction. We assessed soil moisture across the gradient and in response to vegetation removal using ANOVA with sample date, site, treatment, and all interactions as fixed effects. The same model was used to test the effects of vegetation removal compared to controls on light availability at 0.5 m and ground level. For site‐level ambient light availability, we used two‐way ANOVA with sample date, site, and their interaction.

## 3 Results

### 3.1 Factorial common garden experiment

**Figure 2:**
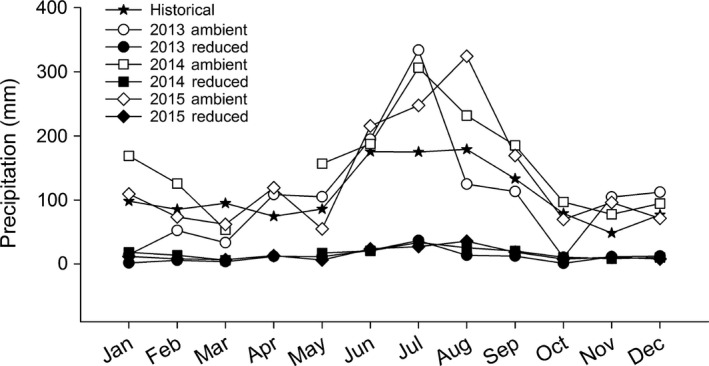
Comparison of 100‐year average precipitation (Historical; weather station ID 083326, Florida Climate Center) in Gainesville, Florida, to (1) monthly ambient precipitation (ambient) at the common garden experiment site from 2013 to 2015 and (2) the amount of precipitation estimated to enter drought treatment plots with an 89% reduction in ambient precipitation (reduced)

Historical, 100‐year average precipitation (weather station ID 083326; Florida Climate Center) and precipitation during the experiment revealed distinct wet and dry seasons (Figure 2). Precipitation during the wet season (May/June–October/November) was above average over 2013–2015 relative to the historic norm.

**Figure 3:**
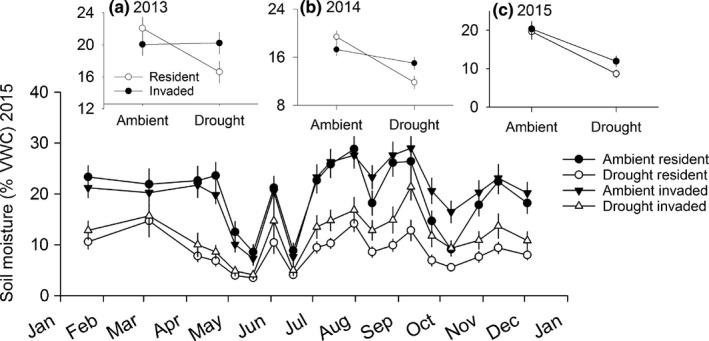
Mean ± *SE* soil moisture (% volumetric water content) from January to December 2015 (main figure) and averaged over the year for 2013 (sampled from July to October), 2014 (sampled from March to December), and 2015 (sampled January to December; insets a–c) in plots exposed to four treatment combinations: (1) ambient precipitation with resident understory species; (2) reduced precipitation (“drought”) with resident understory species; (3) ambient precipitation with resident understory species plus the invader (cogongrass; *Imperata cylindrica*); and (4) drought with resident understory species plus the invader

**Figure 4:**
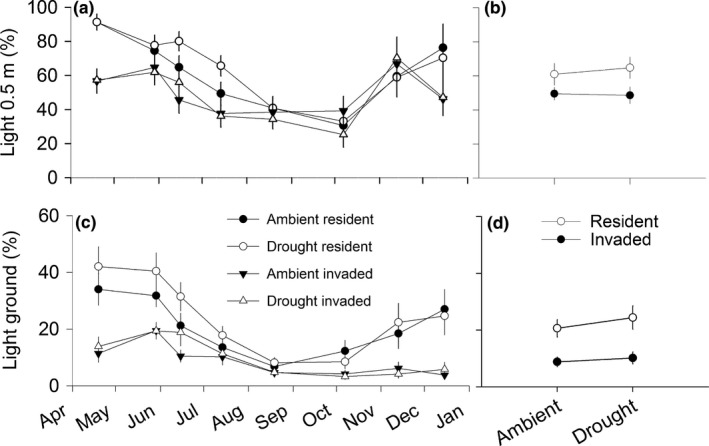
Mean ± *SE* percent light availability (photosynthetically active radiation) at 0.5 m (panels a,b) and ground level (panels c,d) in common garden plots exposed to drought and invasion treatments (defined in Figure 3). Light availability was measured above the vegetation canopy (at 1.4 m) and compared to light levels at 0.5 m and ground level

Control structures created light conditions comparable to the rainout shelters (percent light reduction 33.4 ± *SE* 1.01 and 31.1± *SE* 1.2, respectively; *t*_37_ = 1.5, *p *=* *.14). Drought and invasion significantly affected soil moisture (Appendix S3). In 2015, percent soil moisture was lower in drought plots (drought, 10.3 ± *SE* 0.28; ambient, 20 ± *SE* 0.43; *F*_1,701_ **=* *145; *p *<* *.0001) and higher in invaded plots (resident, 14 ± *SE* 0.45, invaded 16 ± *SE* 0.43; *F*_1,701_ **=* *7.3; *p *=* *.007). The invader consistently maintained higher soil moisture than resident vegetation under drought (2013, drought × invasion, *F*_1,330_ = 3.38; *p *=* *.05; 2014, *F*_1,553_ = 6; *p *=* *.01; 2015, *F*_1,701_ **=* *3.5; *p *=* *.06; Figure 3 insets). Treatment effects on soil moisture varied over time in 2015 (Figure 3, main panel; Appendix S3), with drought effects disappearing after large rain events (e.g., where treatment differences disappear in June 2015, Figure 3) and invasion effects increasing from July onward (Figure 3). Drought had no effect on light at 0.5 m (Appendix S3), but percent light availability was higher in drought (17.3 ± 1.1) than ambient (14.7 ± 0.88) plots at ground level (*F*_1,286_ **=* *8.2; *p *=* *.005). Percent light availability was lower in invaded than resident plots at 0.5 m (*F*_1,286_ **=* *53.5; *p *<* *.0001) and at ground level (*F*_1,286_ **=* *298.1; *p *<* *.0001). Drought and invasion did not interact to affect light (Figure 4 insets), but treatment effects varied in magnitude over time (Figure 4a,b; Appendix S3).

**Figure 5:**
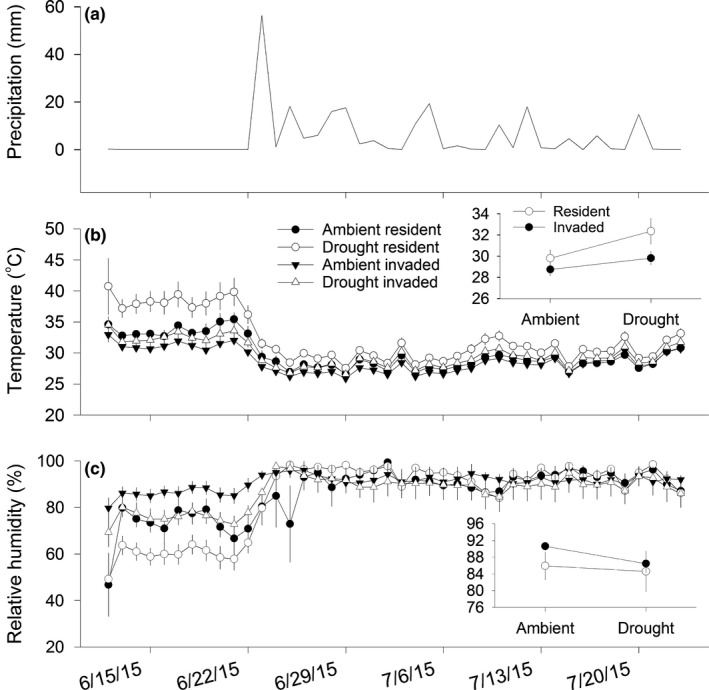
Mean ± *SE* precipitation (a), temperature (b), and humidity (c) during June and July (early to middle of the wet season) in common garden plots exposed to drought and invasion treatments (defined in Figure 3). See Appendix S4 for treatment effects on temperature and humidity during the dry season (November–January)

During the 2015 wet season (Figure 5a; Appendix S3), temperatures were 6% higher (Figure 5b) in drought plots than ambient plots (*F*_1,1026_ **=* *23.6; *p *<* *.0001) and 5.8% lower in invaded plots than resident plots (*F*_1,1026_ **=* *20.9; *p *<* *.0001). Invasion offset drought effects on temperature (Figure 5b, inset; drought × invasion, *F*_1,1026_ **=* *3.4; *p *=* *.06). Drought and invasion did not significantly affect humidity over the entire sampled period (Figure 5c, inset), however prior to a large rain event, humidity was ~17% greater in ambient than drought plots and 22% greater in invaded than resident plots (Figure 5c). For both temperature and humidity, temporal variation stemmed from diminished treatment effects following a June rain event (Figure 5a–c; Appendix S3). See Appendix S4 for dry season treatment effects on temperature and humidity.

### 3.2 Field experiment along natural soil moisture gradient

Percent cover of cogongrass ranged from 50% to 93% (mean ± *SE*, 76.5 ± 4.6) across the gradient in July 2015, while peak biomass ranged from 11 to 26 g/0.0625 m^2^ (mean ± *SE*, 17.7 ± 2.2). Removal of the invader, as measured by percent cover (present, 76.5 ± 3.1; removed 2.26 ± 0.47; *F*_7,32_ **=* *3067; *p *<* *.0001) and biomass (present, 17.7 ± 2.2; removed, 0.004 ± 0.004; *F*_7,32_ **=* *160.5; *p *<* *.0001), was effective and consistent across sites (site × treatment interactions, Appendix S5; site means, Appendix S6). Resident plant cover in July 2015 ranged from 47% to 125% (mean ± *SE*, 72.5 ± 8.7) and biomass from 3.4 to 15.2 g/0.0625 m^2^ (mean ± *SE*, 7.7 ± 1.3). Herbicide reduced cogongrass percent cover (present, 72.5 ± 5.7; removed, 6.8 ± 1.4; *F*_1,36_ **=* *487.3; *p *<* *.0001) and biomass (present, 7.7 ± 0.99; removed, 0.13 ± 0.09; *F*_1,36_ **=* *178.8; *p* < .0001). Removal efficacy varied across sites for percent cover (site × treatment, *F*_8,36_ **=* *2.1; *p *=* *.07; Appendix S6) but not biomass (Appendix S5).

**Figure 6:**
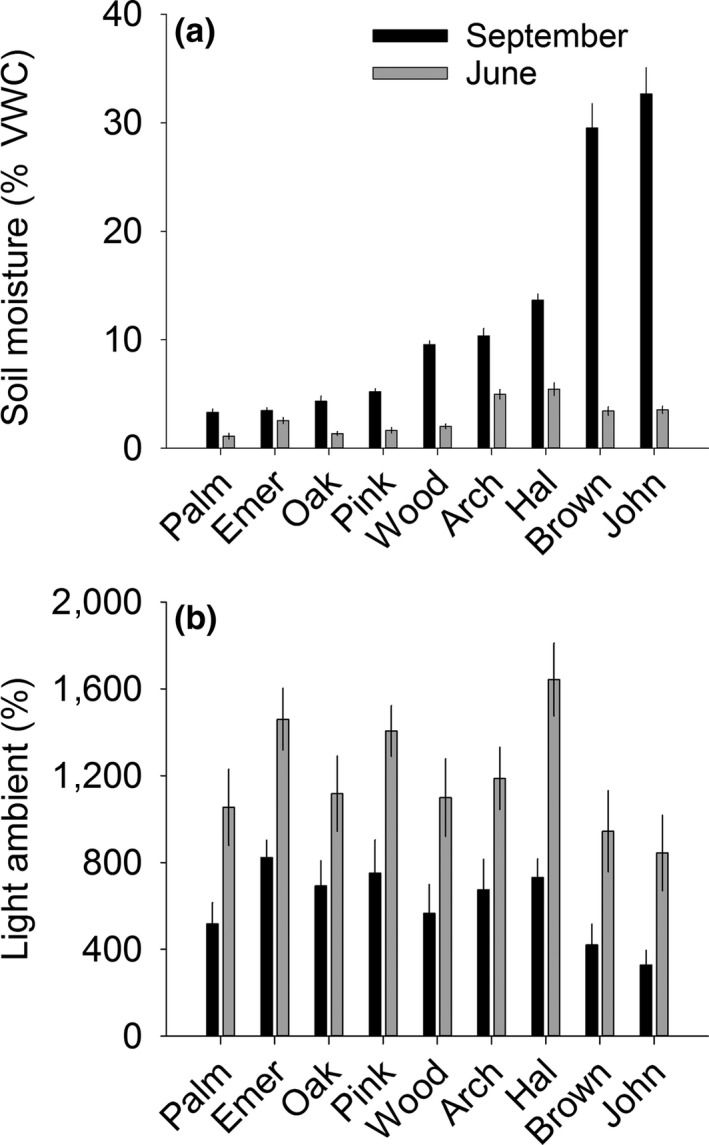
Mean ± *SE* soil moisture (% volumetric water content) (a) and ambient light availability (photosynthetically active radiation) (b) in September 2015 and June 2016 at nine sites occurring along a soil moisture gradient and invaded by cogongrass (*Imperata cylindrica*) in north‐central Florida

**Figure 7:**
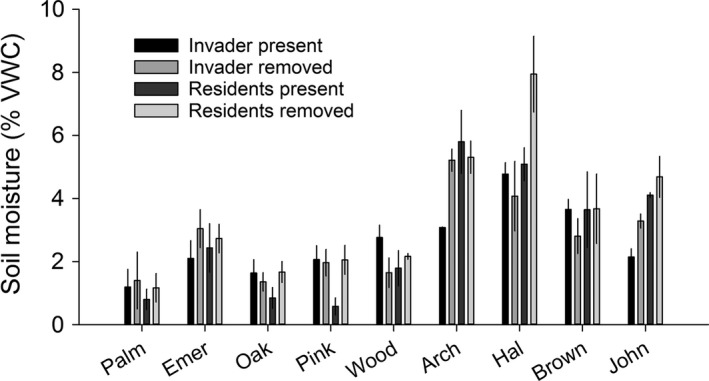
Mean ± *SE* soil moisture (% volumetric water content in September 2015) in response to vegetation removal at nine sites occurring along a soil moisture gradient and invaded by cogongrass (*Imperata cylindrica*) in north‐central Florida. The invader was either left intact (invader present) or removed (invader removed) as was the adjacent uninvaded vegetation (residents present, residents removed)

Soil moisture was higher across sites in September 2015 (12.5%) than June 2016 (2.9%), reflecting the seasonal precipitation of the region. Soil moisture ranged from 3.3% to 32.7% in September and from 1.2% to 5.5% in June (Figure 6a; Appendix S5). The order of sites along the soil moisture gradient shifted between September and June (Figure 6a; sample date × site interaction, *F*_8,164_ **=* *66.6; *p *<* *.0001). Sites differed in ambient light availability at both time points, but their relative differences across the gradient were consistent over time (Figure 6b). Effects of vegetation removal on soil moisture depended on site and time (Appendix S5; see Figure 7 for September trends; see Appendix S7 for June trends). The effect of vegetation removal on light availability also differed by site and time (Appendix S5, interaction terms), but in general, removal of the invader had a larger effect on percent light availability (e.g., for September 2016, present, 42 ± 7.6; removed, 102 ± 7.2) than removal of resident vegetation (present, 93.5 ± 11.2; removed, 100 ± 7.6; see Appendix S8 for light dynamics).

## 4 Discussion

Our methods to evaluate interactions among abiotic and biotic global change drivers with a combination of common garden and field experiments allow for processes to be quantified across spatial scales, and for the experimental and observational studies to ask somewhat different, but mutually informative, questions (Fridley et al., 2007; Stricker, Hagan, & Flory, 2015). Specifically, we are poised to explore an outstanding question in global change research: do complex interactions uncovered in a tightly controlled experiment translate to field conditions? We found that invasion showed the potential to offset drought stress in the common garden but not in the field experiment, while neither experiment suggested that synergistic negative effects via water limitation are inevitable. Going forward, our approach facilitates detailed and long‐term characterization of the ecological responses of plants, arthropods, and microbes to the imposed treatments.

In the common garden experiment, invasion largely offset the rainout shelter effects on soil moisture. This response was unexpected given that the shelters diverted 89% of incoming precipitation, translating into 48% lower soil moisture in drought compared to ambient plots over 2015. Cogongrass has several attributes that might promote soil moisture retention, including high investment in rhizomes that exude water into surrounding soil (Dozier et al., 1998), potentially leading to soil water redistribution (Leffler, Peek, Ryel, Ivans, & Caldwell, 2005), and a dense canopy that may slow evapotranspiration. In support, we found that following a large rain event in June 2015, invaded drought plots maintained higher soil water than resident drought plots. Alternatively, there may be a physiological mechanism to explain these results. For example, the high water‐use efficiency that is characteristic of C4 grasses may promote retention of soil moisture in invaded plots. A critical next step for predicting the longer‐term effects of invasion on ecosystem processes under imposed drought is to measure ecologically relevant traits of the invader and dominant native species, such as stomatal conductance and water‐use efficiency. Given that C4 perennial grasses similar to cogongrass are invasive in regions at high risk for drought (e.g., the arid western US; D'Antonio & Vitousek, 1992), potential amelioration of drought stress in invaded areas could represent an interaction between abiotic and biotic drivers with broad implications for ecosystem response to global change.

Drought had a relatively minor effect on light availability, suggesting that reduced soil moisture did not drastically change resident or invader canopies. However, independent of drought, invaded plots had less light available at 0.5 m and ground level than resident plots, indicative of dense cogongrass stands, which can reduce light and native understory diversity in longleaf pine forests (Brewer, 2008). Another shelter study that compared light attenuation within native and invasive plant canopies exposed to drought (English et al., 2005) revealed that a 50% reduction in summer precipitation in a semi‐arid grassland promoted light availability in native and invaded plots and that, contrary to our study, the native grass reduced light availability more than the invasive grass. The contrast between our findings with a rhizomatous grass and this study on bunchgrasses (English et al., 2005) highlights the challenge of predicting how species with different functional traits will respond to changing conditions in different ecosystems.

On average, the drought treatment also increased temperature during the 2015 wet season, which could exacerbate already‐stressful conditions associated with low soil moisture. The compounding effect of high temperature was more pronounced in resident than invaded plots because, as with soil moisture, invasion offset the effect of drought on high temperatures. A parallel pattern also occurred with humidity prior to a large rain event in June, after which the offsetting effect of invasion on low humidity disappeared. Taken together, these findings reveal that the invader mitigated a constellation of abiotic conditions that can elicit plant water stress—namely, low soil moisture, high temperatures, and low humidity. Such interactions between drought and plant invasions may be particularly important in high‐precipitation, subtropical regions because stress from less‐frequent precipitation events may be more pronounced in mesic than xeric ecosystems (Beier et al., 2012; Knapp et al., 2008). Given that the invader's potential to mitigate drought stress also was associated with reduced light availability, we expect that species with different resource‐use strategies will vary in how they respond to these altered conditions. For example, in longleaf pine ecosystems, mesophytic species tolerant of low light, including some non‐native invaders like Chinese tallow (*Triadica sebifera*), could establish. Alternatively, shade‐intolerant species that colonize canopy gaps in the understory (McGuire et al., 2001) might become less successful under invaded conditions, even if soil moisture stress is alleviated. Thus, the identity of the species that become extirpated or established in these altered habitats affected by climate change and invasions, and the resulting effect of species re‐shuffling on native diversity, will largely determine the degree to which desirable ecosystem processes are retained over the longer term.

Because the relationship between precipitation and soil moisture can vary based on shelter design and local conditions, it is difficult to compare our study with others that do not report shelter effects on soil moisture. In particular, shelter height, area, and construction materials, as well as plant community composition and edaphic factors, can mediate microclimate and thus evapotranspiration and soil moisture (Cherwin & Knapp, 2012; Fay et al., 2000; Vogel et al., 2013; Yahdjian & Sala, 2002). Further, even when shelters similarly reduce soil moisture, it is challenging to compare the ecological meaning of reductions among locations that have distinct ecological and evolutionary histories. To facilitate cross‐site comparisons, we recommend that studies report the temporal and spatial effects of shelters on soil moisture, as well as a suite of easily compared response variables measured at different levels of organization (e.g., from specific leaf area at the scale of individuals to net primary production at the scale of ecosystems). We also emphasize that community and ecosystem responses to imposed treatments must be interpreted in the context of long‐term precipitation patterns for the study region (Knapp et al., 2016). In our case, the first 3 years of this long‐term experiment occurred during years with above‐average summer precipitation relative to the historic norm, despite the region having experienced exceptional drought in the years preceding the experiment (e.g., 2006–2008; Wang et al., 2010). Long‐term experiments that span both wet and dry years are thus critical to evaluating community and ecosystem responses to extreme precipitation events (Knapp et al., 2016).

We experimentally removed vegetation from sites along a natural soil moisture gradient to infer the effects of drought and invasion on multiple factors also measured in the common garden. Only in recent years have invader impacts started to be well quantified (Barney, Tekiela, Dollete, & Tomasek, 2013; Pyšek et al., 2012; Stricker et al., 2015; Vilà et al., 2011) and we are not aware of any removal experiments assessing invader effects along an environmental gradient in the context of climate change. Further, few studies compare removal of invaded vegetation to removal of resident vegetation, which is necessary for disentangling effects of the invasion from disturbances created by vegetation removal (Stricker et al., 2015). Invader cover was at least 50% at all field experiment sites, a value typical of many invader impact studies (Barney et al., 2013), but was often higher, thereby capturing a range of invasion densities. Glyphosate was effective in removing cogongrass, likely in part because cogongrass grows rapidly and effectively translocates the systemic herbicide following foliar application (Enloe et al., 2013). While glyphosate also was effective in removing resident vegetation, woody species maintained appreciable cover (up to 20%) at some sites when the herbicide was applied. Such variation necessitates regularly assessing plant cover to account for variable removal efficacy.

Soil moisture at the driest gradient sites was lower than at any time in the common garden, even under rainout shelters, suggesting that the gradient captured conditions as severe or more severe than those imposed in the garden. The seasonal precipitation dynamics at the garden experiment location also manifested in the field, with all sites exhibiting higher soil moisture at the end of the rainy season (September) than at the end of the dry season (June). The gradient in soil moisture was more distinct in September than in June and sites ranked differently along the gradient at the two time points. Our sites also had a gradient in ambient light availability but, unlike soil moisture, the relative differences among sites were consistent across time. Thus, some site characteristics are likely to be more (soil moisture) or less (ambient light) temporally variable than others, indicating that the timing, duration, and frequency of data collection must be designed to capture differences in abiotic conditions that potentially underlie community and ecosystem responses.

At most gradient sites, soil moisture did not differ among plots with intact resident and invaded vegetation. Whether vegetation removal resulted in greater, less, or no effect on soil moisture depended on season and site, but not vegetation type. These findings were contrary to our expectations that (1) removal would either increase soil moisture due to lower transpiration without the abundant invader, or decrease soil moisture due to greater evaporation from the bare soil surface; and (2) there would be differences in soil moisture responses associated with the unique characteristics of invaded compared to resident vegetation (as in the common garden experiment). Instead, amidst variation from other factors in the field, invasion did not uniquely or consistently affect soil moisture. In contrast, while removal effects on light availability also varied by season and site, light availability was generally greater when the invader was removed than when resident vegetation was removed, consistent with the garden experiment. These results highlight the importance of diligently characterizing site conditions so that they can be included as co‐variates in statistical analyses, as variation from factors other than the responses of interest may mediate community and ecosystem responses to field manipulations.

In conclusion, our approach of coupling a tightly controlled common garden experiment at a single site with a field experiment conducted across a natural environmental gradient provides a mechanistic, yet realistic, method for evaluating interactions among global change drivers. Such coupled experiments are necessary to unravel the potentially complex relationships between climate change and other global change drivers such as plant invasions or shifts in the distribution or abundance of plant communities due to land use change. We emphasize that consideration of both spatial and temporal variation is necessary to provide sufficient data to enhance models for predicting responses to global change. As such, similar complementary approaches as we describe here, undertaken by coordinated networks of researchers in different locations (Fraser, Carlyle, White, & Beierkuhnlein, 2013; Knapp et al., 2016), will have the most power to predict how multiple drivers of global change might interact to affect communities and ecosystems.

## Conflict of Interest

None declared.

## Data Accessibility

The raw data describing soil moisture, light, temperature, humidity, and plant cover in the experimental treatments are available via Dryad, doi: http://dx.doi.org/10.5061/dryad.742vb/1.
