# Supplementary material for: Methods to test the interactive effects of drought and plant invasion on ecosystem structure and function using complementary common garden and field experiments
Source: Ecol Evol. 2017 Feb 5;7(5):1442–52. doi: 10.1002/ece3.2729 (PMC5330907; doi:10.1002/ece3.2729)
Supplement: Supplementary file 1 [file ECE3-7-1442-s001.docx]

Appendix S1: Methods and results to determine shelter edge effects on temperature and humidity

Hygrochron^TM^ ibutton sensors (model DS1923; Embedded Data Systems, LLC; Lawrenceburg, KY) were arranged in perpendicular transects oriented in the north-south and east-west directions across the plot centers (sub-sample of n = 4 plots x 3 blocks = 12 plots; sensors were deployed for one week per block from mid-August to mid-September 2015). We used mixed model ANOVA in SAS to test for shelter edge effects (SAS v. 9.4, SAS Institute, Cary, NC). The fixed effect was the location of each ibutton sensor in the plots (assigned as 1-5 running from the north-to-south or east-to-west edges of the plot, with 3 indicating the plot center) and the random effect was block. Square-root transformation greatly improved normality and homogeneity of variance for humidity but not temperature. However, the sample size for the temperature measurements was large enough (N > 50) to support a parametric approach (Pearson 1931; Glass *et al.* 2012). When significant main effects were present, differences of least square means were used to estimate pairwise differences.

The only microclimatic variable that showed significant variation among sensor locations was temperature in the E-W direction across the shelter (Table 1). However, these differences did not indicate an edge effect (i.e., they did not consistently increase or decrease from the center to the edges of the shelters). Additionally, while statistically significant, the differences in temperature were quite small and not likely to have a strong effect on microclimatic conditions experienced by plants at the within-plot scale.

Table 1. Statistical results of mixed model ANOVA testing for shelter edge effects in a common garden experiment using rainout shelters to simulate drought.

|  | Temperature in $^{\circ}$C  Mean (SE) | | % Relative Humidity  Mean (SE) | |
| --- | --- | --- | --- | --- |
| Sensor Location | NS direction | *EW direction | NS direction | EW direction |
| 1: edge | 25.14 | ^a^25.09 (0.67) | 93.60 | 88.57 (3.92) |
| 2: edge-center | 25.22 | ^abc^25.18 (0.67) | 90.98 | 88.99 (3.92) |
| 3: center | 25.03 | ^ac^25.03 (0.67) | 86.60 | 86.58 (3.79) |
| 4: edge-center | 25.30 | ^ac^25.00 (0.67) | 91.20 | 90.65 (3.79) |
| 5: edge | 25.44 | ^b^25.52 (0.67) | 89.31 | 83.52 (4.06) |
| *F*-value_(ndf, ddf)_ | 1.44_(4,38)_ | 2.99_(4,49)_ | 2.11_(4,38)_ | 0.62_(4,49)_ |
| *P*-value | 0.24 | 0.03 | 0.10 | 0.65 |

*Different letters before means indicate significant differences among sensor locations

References Cited

Glass, G. V, Peckham, P.D. & Sanders, J.R. (2012). Consequences of Failure to Meet Assumptions Underlying the Fixed Effects Analyses of Variance and Covariance Author ( s ): Gene V . Glass , Percy D . Peckham and James R . Sanders Reviewed work ( s ): Source : Review of Educational Research , Vol . 42 , N. *Review of Educational Research*, **42**, 237–288.

Pearson, E.S. (1931). Biometrika Trust. *Biometrika*, **38**, 11–25.
