# Supplementary material for: Methods to test the interactive effects of drought and plant invasion on ecosystem structure and function using complementary common garden and field experiments
Source: Ecol Evol. 2017 Feb 5;7(5):1442–52. doi: 10.1002/ece3.2729 (PMC5330907; doi:10.1002/ece3.2729)
Supplement: Supplementary file 2 [file ECE3-7-1442-s002.docx]

Appendix S2: Locations and soil characteristics of sites used in the field gradient experiments. Sites are ordered from lowest to highest soil moisture content following the 2015 wet season (see Figure 6 in the manuscript). Some sites spanned multiple soil series and texture classes.

| Site | Latitude | Longitude | Series* | Texture Class | Drainage Class |
| --- | --- | --- | --- | --- | --- |
| Palm | 81°59.821W | 29°05.453N | Astatula | Sand | Excessively drained |
|  |  |  | Candler | Sand | Excessively drained |
| Emerald | 82°00.195W | 29°05.821N | Candler | Sand | Excessively drained |
| Oak Road | 82°00.450W | 29°04.051N | Apopka | Sand | Well drained |
|  |  |  | Candler | Sand | Excessively drained |
| Pinkoson | 82°35.309W | 29°36.031N | Candler | Sand | Excessively drained |
|  |  |  | Millhopper | Sand | Moderately well drained |
| Wood | 82°26.353W | 29°49.251N | Millhopper | Sand | Moderately well drained |
|  |  |  | Tavares | Sand | Moderately well drained |
| Archer | 82°30.949W | 29°32.368N | Arredondo | Fine sand | Well drained |
|  |  |  | Kendrick | Sand | Well drained |
|  |  |  | Bonneau | Fine sand | Moderately well drained |
| Halbrook | 82°25.358W | 29°54.146N | Millhopper | Sand | Moderately well drained |
| Brown | 82°20.109W | 29°21.633N | Flemington | Loamy sand | Poorly drained |
|  |  |  | Micanopy | Fine sand | Somewhat poorly drained |
| Johnson | 82°05.784W | 29°33.252N | Sparr | Fine sand | Somewhat poorly drained |
|  |  |  | Pottsburg | Sand | Poorly drained |

*Soil characteristics from the USDA Natural Resources Conservation Service Web Soil Survey (http://websoilsurvey.sc.egov.usda.gov/App/WebSoilSurvey.aspx; accessed November 2016)
