# Supplementary material for: Methods to test the interactive effects of drought and plant invasion on ecosystem structure and function using complementary common garden and field experiments
Source: Ecol Evol. 2017 Feb 5;7(5):1442–52. doi: 10.1002/ece3.2729 (PMC5330907; doi:10.1002/ece3.2729)
Supplement: Supplementary file 3 [file ECE3-7-1442-s003.docx]

Appendix S3. Results for mixed model ANOVAs testing the effects of drought, invasion, date and their interactions on several environmental variables in the common garden experiment.

|  | Model Effect | *F*-value_(ndf, ddf)_ | *P*-value |
| --- | --- | --- | --- |
| Soil Moisture (Vol. Water Content) | Drought  Invasion  Drought x invasion  Date  Drought x date  Invasion x date | 145_(1,701)_  7.3_(1,701)_  3.5_(1,701)_  104_(18,701)_  7.7_(18,701)_  2.2_(18,701)_ | < 0.0001  0.007  0.06  < 0.0001  < 0.0001  0.002 |
| Photosynthetically active radiation  Upper canopy (0.5 m)      Ground level (0 m) | Drought  Invasion  Drought x invasion  Date  Drought x date  Invasion x date  Drought  Invasion  Drought x invasion  Date  Drought x date  Invasion x date | 0.63_(1,286)_  53.3_(1,286)_  1.26_(1,286)_  44.6_(7,286)_  1.92_(7,286)_  9.29_(7,286)_  8.2_(1,286)_  298.1_(1,286)_  0.65_(1,286)_  63.4_(7,286)_  2.78_(7,286)_  11.3_(7,286)_ | 0.43  < 0.0001  0.26  < 0.0001  0.066  < 0.0001  0.005  < 0.0001  0.42  < 0.0001  0.008  < 0.0001 |
| Temperature $℃$ | Drought  Invasion  Drought x invasion  Date  Drought x date  Invasion x date | 23.6_(1,1026)_  20.9_(1,1026)_  3.4_(1,1026)_  97.4_(41,1026)_  2.5_(41,1026)_  1.8_(41,1026)_ | < 0.0001  < 0.0001  0.06  < 0.0001  < 0.0001  0.002 |
| Percent Relative Humidity | Drought  Invasion  Drought x invasion  Date  Drought x date  Invasion x date | 2.1_(1,1026)_  2.4_(1,1026)_  1.2_(1,1026)_  7.8_(41,1026)_  1.4_(41,1026)_  1.9_(41,1026)_ | 0.15  0.12  0.27  < 0.0001  0.055  0.0005 |
