# Supplementary material for: Methods to test the interactive effects of drought and plant invasion on ecosystem structure and function using complementary common garden and field experiments
Source: Ecol Evol. 2017 Feb 5;7(5):1442–52. doi: 10.1002/ece3.2729 (PMC5330907; doi:10.1002/ece3.2729)
Supplement: Supplementary file 4 [file ECE3-7-1442-s004.docx]

Appendix S4. Statistical methods and results describing the effects of drought and invasion treatments on temperature and humidity during the dry season at the common garden experiment site. Compare with wet-season results presented in the manuscript text.

We used mixed model ANOVA with the fixed effects of drought status (ambient precipitation or imposed drought), invasion status (resident understory species only, or resident understory species invaded by cogongrass, *Imperata cylindrica*), a drought x invasion interaction, sampling date, and sampling date x treatment interactions. We included block as a random effect and treated plots as a repeated measure across sampling dates (using the “subject” command in the “repeated” statement with an autoregressive covariance structure). Inspection of model residual versus quantile plots and the distribution of model residuals versus predicted values indicated relatively good model fit without the need to transform the variables. Statistical analyses were conducted in SAS v. 9.4 (SAS Institute, Cary, NC).

Precipitation during the dry-season sampling event was 44.7% of that available during to the 2015 wet-season sampling event (compare supplementary Figure 1a below to Figure 2 in the manuscript). Temperatures in drought plots were ~4% higher than temperatures in ambient plots (Figure 1b inset; *F*-value_(ndf, ddf)_ = 2.2_(1,1032)_; *P* = 0.04) while temperature in invaded plots was 5% lower than temperature in resident plots (*F*-value_(ndf, ddf)_ = 61.1_(1,1032)_; *P* < 0.0001). The effects of drought (drought x time, *F*-value_(ndf, ddf)_ = 1.7_(42,1032)_; *P* = 0.003) and invasion (invasion x time, *F*-value_(ndf, ddf)_ = 2.2_(42,1032)_; *P* < 0.0001) on temperature changed over the course of the ~6-week sampling event, with treatment effects becoming less pronounced after rain events (Figure 1a,b). In contrast to what we observed during the wet season, invasion did not disproportionately offset the effects of drought on temperature relative to resident plots (Figures 1b,c insets; drought x invasion, *F*-value_(ndf, ddf)_ = 0.92_(1,1032)_; *P* = 0.34).

Drought had no overall effect on humidity, decreasing it by only 1.5% relative to ambient conditions (Figure 1c inset). Drought effects on humidity did change over time (drought x date, *F*-value_(ndf, ddf)_ = 1.6_(42,1032)_; *P* = 0.01; Appendix S2, Figure 1c), becoming less pronounced after rain events (Figure 1 a,b). Invasion increased humidity by ~4% (Figure 1 inset *F*-value_(ndf, ddf)_ = 20.9_(1,1026)_; *P* < 0.0001) and its effect was consistent across drought and ambient plots (Figure 1 inset; drought x invasion, *F*-value_(ndf, ddf)_ = 0.01_(1,1032)_; *P* = 0.98) and over time (Figure 1c; invasion x date interaction, *F*-value_(ndf, ddf)_ = 0.93_(42,1032)_; *P* = 0.61).
