# Supplementary material for: Methods to test the interactive effects of drought and plant invasion on ecosystem structure and function using complementary common garden and field experiments
Source: Ecol Evol. 2017 Feb 5;7(5):1442–52. doi: 10.1002/ece3.2729 (PMC5330907; doi:10.1002/ece3.2729)
Supplement: Supplementary file 5 [file ECE3-7-1442-s005.docx]

Appendix S5. Results for ANOVA models describing variation in soil moisture, light availability, and vegetation removal efficacy in the field experiment along a natural soil moisture gradient. The effects of vegetation removal treatments were not included in the models for ambient light availability at each site because these measurements were taken above the understory canopy.

|  | Model Effect | *F*-value_(ndf, ddf)_ | *P*-value |
| --- | --- | --- | --- |
| Removal Efficacy  Resident percent cover  Resident biomass  Invader percent cover  Invader biomass  Soil Moisture (Vol. Water Content) | Site  Treatment  Site x trt  Site  Treatment  Site x trt  Site  Treatment  Site x trt  Site  Treatment  Site x trt  Sample date  Site  Treatment  Sample date x site  Sample date x trt  Site x trt | 9.5_(8,36)_  487.3_(1,36)_  2.1_(8,36)_  3.15_(8,36)_  178.8_(1,36)_  1.7_(8,36)_  8.3_(8,36)_  1935_(1,36)_  1.5_(8,36)_  0.54_(8,36)_  185.8_(1,36)_  0.49_(8,36_  1002.7_(1,164)_  138.8_(8,164)_  0.85_(3,164)_  66.6_(8,164)_  5.2_(3,164)_  2.0_(24,164)_ | <0.0001  < 0.0001  0.07  0.008  < 0.0001  0.13  <0.0001  < 0.0001  0.21  0.82  < 0.0001  0.85  < 0.0001  < 0.0001  0.47  < 0.0001  0.002  0.007 |
| PAR  Ambient (above canopy)  0.5 m  ground | Sample date  Site  Sample date x site  Sample date  Site  Treatment  Sample date x trt  Site x trt  Sample date  Site  Treatment  Sample date x trt  Site x trt | 83.2_(1,194)_  4.5_(8,194)_  0.65_(8,194)_  7.6_(1,172)_  1.1_(8,172)_  4.3_(3,172)_  6.9_(3,172)_  1.49_(24,172)_  16.2_(1,172)_  3.8_(8,172)_  67.7_(3,172)_  6.8_(3,172)_  2.1_(24,172)_ | < 0.0001  < 0.0001  0.73  0.007  0.39  0.006  0.0002  0.08  < 0.0001  0.0004  < 0.0001  0.0003  0.003 |
|  |  |  |  |
