# Supplementary material for: Methods to test the interactive effects of drought and plant invasion on ecosystem structure and function using complementary common garden and field experiments
Source: Ecol Evol. 2017 Feb 5;7(5):1442–52. doi: 10.1002/ece3.2729 (PMC5330907; doi:10.1002/ece3.2729)
Supplement: Supplementary file 6 [file ECE3-7-1442-s006.docx]

Appendix S6. Mean $\pm$ SE percent cover (first entry per site) and grams biomass (second entry per site) in plots where the invader (cogongrass, *Imperata cylindrica*) and resident vegetation were either left intact or removed with a broad-spectrum herbicide. Sites occurred along a soil moisture gradient (ordered from low to high as in manuscript Figure 6) in north-central Florida.

| Site | Invader present | Invader removed | Resident present | Resident removed |
| --- | --- | --- | --- | --- |
| Palm | 78.3 $\pm$ 6  11.1 $\pm$ 1.8 | 0.67 $\pm$ 0.33  0 $\pm$ 0 | 58.8 $\pm$ 4.5  8.2 $\pm$ 1.4 | 2 $\pm$ 0  0 $\pm$ 0 |
| Emer | 73.3 $\pm$ 6.7  17 $\pm$ 0.94 | 1 $\pm$ 0  1$\pm$ 0 | 65.7 $\pm$ 10.2  8.4 $\pm$ 2.4 | 1 $\pm$ 0  0 $\pm$ 0 |
| Oak | 50 $\pm$ 13.2  12.8 $\pm$ 3.9 | 0.33 $\pm$ 0  0 $\pm$ 0 | 51.7 $\pm$ 21.9  3.4 $\pm$ 2.7 | 1 $\pm$ 0.58 0 $\pm$ 0 |
| Pink | 65 $\pm$ 7.6  15.6 $\pm$ 4.2 | 2.3 $\pm$ 1.3  0 $\pm$ 0 | 93.3 $\pm$ 11.7  6.3 $\pm$ 0.56 | 3.7 $\pm$ 1.5  0 $\pm$ 0 |
| Wood | 76.6 $\pm$ 3.3  22.7 $\pm$ 9.8 | 2.3 $\pm$ 1.3  0 $\pm$ 0 | 47 $\pm$ 8.5  5.6 $\pm$ 2.9 | 11 $\pm$ 3  0.24 $\pm$ 0.24 |
| Arch | 91.7 $\pm$ 1.7  13.2 $\pm$ 8.1 | 5 $\pm$ 0  0 $\pm$ 0 | 125 $\pm$ 5.3  15.2 $\pm$ 1.9 | 21.7 $\pm$ 3.3  0.92 $\pm$ 0.66 |
| Hal | 93.3 $\pm$ 1.7  26.3 $\pm$ 16.3 | 6.7 $\pm$ 1.7  0.04 $\pm$ 0 | 92 $\pm$ 3  12.7 $\pm$ 2.8 | 10.3 $\pm$ 2.9  0 $\pm$ 0 |
| Brown | 88.3 $\pm$ 4.4  19.7 $\pm$ 0.96 | 1 $\pm$ 0  0 $\pm$ 0 | 70.3 $\pm$ 13  6.6 $\pm$ 3.1 | 8 $\pm$ 2.3  0 $\pm$ 0 |
| John | 71.7 $\pm$ 1.7  20.6 $\pm$ 3.9 | 1 $\pm$ 0  0 $\pm$ 0 | 48.7 $\pm$ 9.5  3 $\pm$ 1.8 | 2.7 $\pm$ 1.8  0 $\pm$ 0 |
