# Supplementary material for: Methods to test the interactive effects of drought and plant invasion on ecosystem structure and function using complementary common garden and field experiments
Source: Ecol Evol. 2017 Feb 5;7(5):1442–52. doi: 10.1002/ece3.2729 (PMC5330907; doi:10.1002/ece3.2729)

Appendix S7. Mean $\pm$ SE soil moisture (% volumetric water content in June 2016) in response to vegetation removal at nine sites occurring along a soil moisture gradient and invaded by cogongrass (*Imperata cylindrica*) in north-central Florida. The invader was either left intact (Invader present) or removed (Invader removed) as was the adjacent uninvaded vegetation (Residents present, Residents removed). Sites are ordered as in manuscript Figure 7.


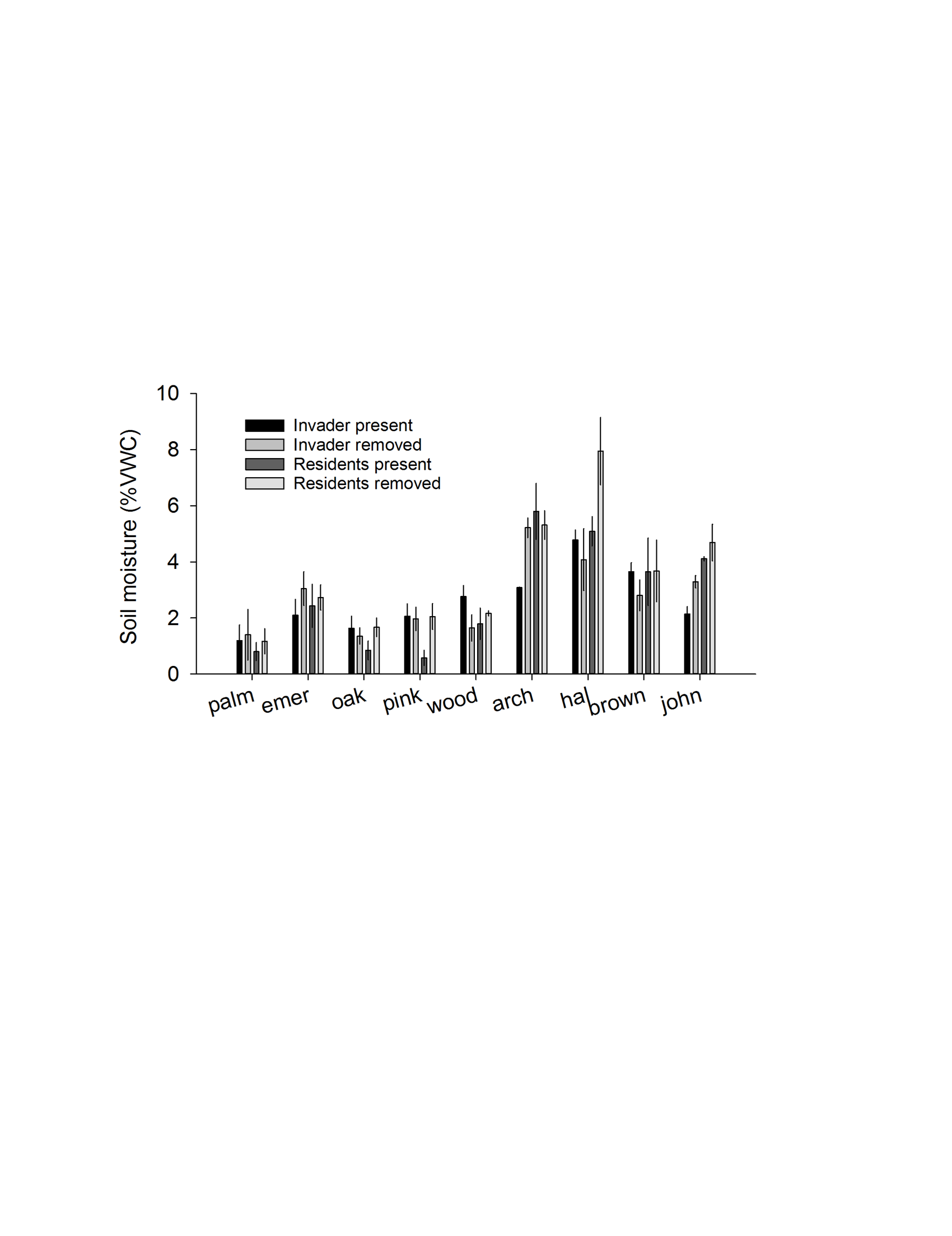

Supplement: Supplementary file 7 [file ECE3-7-1442-s007.docx]
