# Supplementary material for: Methods to test the interactive effects of drought and plant invasion on ecosystem structure and function using complementary common garden and field experiments
Source: Ecol Evol. 2017 Feb 5;7(5):1442–52. doi: 10.1002/ece3.2729 (PMC5330907; doi:10.1002/ece3.2729)
Supplement: Supplementary file 8 [file ECE3-7-1442-s008.docx]

Appendix S8. Mean $\pm$ SE percent light (photosynthetically active radiation) available at mid-canopy height (0.5 m, panels a and c) and ground level (panels b and d) relative to ambient conditions (measured above the vegetation canopy at 1.5 m). Measurements were made in September 2015 and June 2016 to assess light levels within invaded (by cogongrass, *Imperata cylindrica*) and resident vegetation canopies at nine sites occurring along a soil moisture gradient in north-central Florida. The invader was either left intact (Invader present) or removed (Invader removed) as was the adjacent uninvaded vegetation (Residents present, Residents removed). Sites are ordered as in manuscript Figure 6.
